# Basement membrane proteins modulate cell migration on bovine pericardium extracellular matrix scaffold

**DOI:** 10.1038/s41598-021-84161-5

**Published:** 2021-02-25

**Authors:** Qi Xing, Mojtaba Parvizi, Manuela Lopera Higuita, Leigh G. Griffiths

**Affiliations:** Department of Cardiovascular Diseases, Mayo Clinic, Stabile 4-58, 200 First Street, Rochester, MN 55905 USA

**Keywords:** Biomaterials, Regenerative medicine, Tissue engineering

## Abstract

Native bovine pericardium (BP) exhibits anisotropy of its surface ECM niches, with the serous surface (i.e., parietal pericardium) containing basement membrane components (e.g., Laminin, Col IV) and the fibrous surface (i.e., mediastinal side) being composed primarily of type I collagen (Col I). Native BP surface ECM niche anisotropy is preserved in antigen removed BP (AR-BP) extracellular matrix (ECM) scaffolds. By exploiting sideness (serous or fibrous surface) of AR-BP scaffolds, this study aims to determine the mechanism by which ECM niche influences human mesenchymal stem cells (hMSCs) migration. Human mesenchymal stem cells (hMSC) seeding on serous surface promoted more rapid cell migration than fibrous surface seeding. Gene analysis revealed that expression of integrin α_3_ and α_11_ were increased in cells cultured on serous surface compared to those on the fibrous side. Monoclonal antibody blockade of α_3_β_1_ (i.e., laminin binding) inhibited early (i.e. ≤ 6 h) hMSC migration following serous seeding, while having no effect on migration of cells on the fibrous side. Blockade of α_3_β_1_ resulted in decreased expression of integrin α_3_ by cells on serous surface. Monoclonal antibody blockade of α_11_β_1_ (i.e., Col IV binding) inhibited serous side migration at later time points (i.e., 6–24 h). These results confirmed the role of integrin α_3_β_1_ binding to laminin in mediating early rapid hMSCs migration and α_11_β_1_ binding to Col IV in mediating later hMSCs migration on the serous side of AR-BP, which has critical implications for rate of cellular monolayer formation and use of AR-BP as blood contacting material for clinical applications.

## Introduction

Bovine pericardium (BP) derived biomaterials have been widely used in a variety of surgical applications since its first introduction in clinical practice^1^. Glutaraldehyde-fixed BP (GFBP) for example is currently widely used clinically for bioprosthetic heart valve fabrication and arterial patches. GFBP has many advantages compared to synthetic materials, such as off-shelf availability, easy handling, and reduced suture bleeding^2^. Additionally, glutaraldehyde fixation can prevent hyperacute and acute immune response by masking xenoantigens in BP. However, persistent presence of xenoantigens in GFBP results in chronic immune-mediated degeneration and subsequent calcification^3,4^. Additionally, residual glutaraldehyde in GFBP have also been linked with toxicity towards repopulating recipient cells^5,6^. Consequently, despite its short term benefits GFBP exhibits limited long term host cell repopulation, tissue integration and biomaterial remodeling. The limitation of GFBP can be potentially overcome by development of unfixed BP extracellular matrix (ECM) scaffolds which have been processed to reduce the antigenic content of the biomaterial, rendering it minimally antigenic and compatible with recipient non-immune cellular repopulation.

An ideal decellularization method should eliminate candidate tissue xenoantigens, while maintaining native ECM structure–function properties. A variety of decellularization approaches have been explored to remove antigenic components from native BP, including sodium dodecyl sulfate (SDS), TritonX-100, and trypsin^7^. It has been reported that SDS-decellularization can achieve complete acellularity; however, ECM structure–function properties of such scaffolds are significantly altered due to the denaturing properties of this ionic denaturing detergent^7–9^.. Disruption of the native ECM, especially basement membrane integrity, can negatively impact cell–matrix interactions altering cell phenotype, proliferation, survival and migration behavior^7^. Native BP has two distinct surfaces: (1) the parietal pericardial (i.e., serous) surface consists of an endothelial cell monolayer and underlying basement membrane; (2) the mediastinal (i.e., fibrous) surface is composed of connective tissue. A stepwise, solubilization based antigen-removal (AR) approach has been shown to significantly reduced antigenicity, while maintaining native BP ECM structure–function properties, thereby providing a significant advance in the field when compared to traditional decellularization methods^9–11^. In particular, the major basement membrane proteins such as laminin and collagen IV (Col IV) are preserved on serous side of BP-AR scaffolds, while the fibrous side exhibits a predominantly Col I surface composition. Consequently the surface ECM niche anisotropy of AR-BP scaffolds provides a unique opportunity to examine the effect of basement membrane vs. non-basement membrane surface on repopulating cell behavior.

Cell migration on ECM scaffolds is crucial in many biological processes including vascular tissue endothelialization and tissue regeneration. Human mesenchymal stem cells (hMSCs) derived from bone marrow has been reported to migrate/adhere to area’s of tissue injury or implanted grafts, and contribute to tissue regeneration^12^. It is suggested for instance that remodeling of vascular ECM scaffolds is dependent on adhesion, migration, proliferation and differentiation of such circulating cells. Previous experiments have shown that circulating progenitor cells, including hMSCs, contribute to endothelialization of the luminal surface of vascular ECM scaffolds^13^. The ability of hMSCs to differentiate into vascular cells demonstrates the important role of this cell population in vascular tissue regeneration^14^. However, the ECM niche factors which affect hMSC repopulation rate remain unknown. Determination of the ECM niche factors responsible for modulation of hMSCs migration on ECM scaffolds is critical to inform which side of such biomaterials should face the vascular lumen to enhance rate of luminal cellular repopulation.

In this work, hMSCs were employed as model cells to study in vitro cell migration on the serous versus the fibrous surfaces of BP scaffolds. It was previously shown that the different ECM niches of AR-BP scaffolds affected hMSCs proliferation and cytokine secretion^15^. We hypothesize that the basement membrane proteins laminin and collagen IV on the serous surface promote X–Y hMSCs migration across the scaffold surface, while absence of basement membrane components favors Z-direction migration. We further hypothesize that higher levels of cell migration is mainly mediated via interaction between integrin α_3_β_1_ and laminin. We evaluated the effect of BP-AR ECM surface niche on hMSC X–Y migration, Z-direction penetration, and proliferation into AR-BP, and integrin gene expression. To further define the mechanism of ECM niche dependent migration, we investigated the effect of integrin α_3_β_1_ and α_11_ chain blockade on ECM niche dependent migration and integrin gene expression.

## Results

### AR preserves native BP surface morphology and ECM component anisotropy

**Figure 1:**
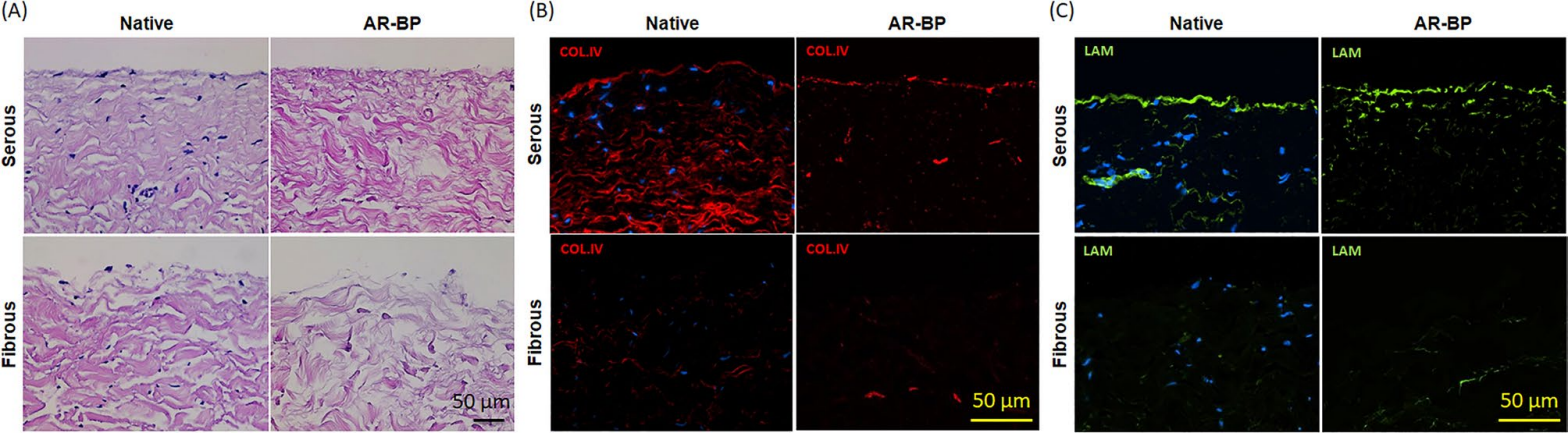
Hematoxylin & eosin (HE) and basement membrane proteins staining of serous and fibrous surface of native BP and AR-BP. (**A**) HE staining demonstrating that AR scaffolds are completely acellular, while retaining native BP collagen organization. (**B**) Collagen IV immunofluoresence staining demonstrating that AR-BP scaffolds retain native Col IV staining on the serous surface and vascular lumen. Fibrous surface of both native and AR-BP scaffolds does not contain Col IV. (**C**) Laminin immunofluoresence staining demonstrating retention of native tissue laminin content and organization, on the serous surface and vascular lumen of AR-BP scaffolds.

Effect of AR on the native ECM niche anisotropy of BP was investigated via histology and immunofluorescence. Hematoxylin and eosin (H&E) staining of native BP and AR-BP showed that collagen organization of both the serous and fibrous sides is maintained after AR (Fig. 1A). Immunofluorescence staining for Col IV (Fig. 1B) and Laminin (Fig. 1C) demonstrated that only serous surface of native BP contained these two basement membrane protein. Col IV and Laminin content and organization were preserved in BP-AR scaffolds.

### Effect of surface ECM niche anisotropy on X–Y planar cell migration and Z-direction cell penetration

**Figure 2:**
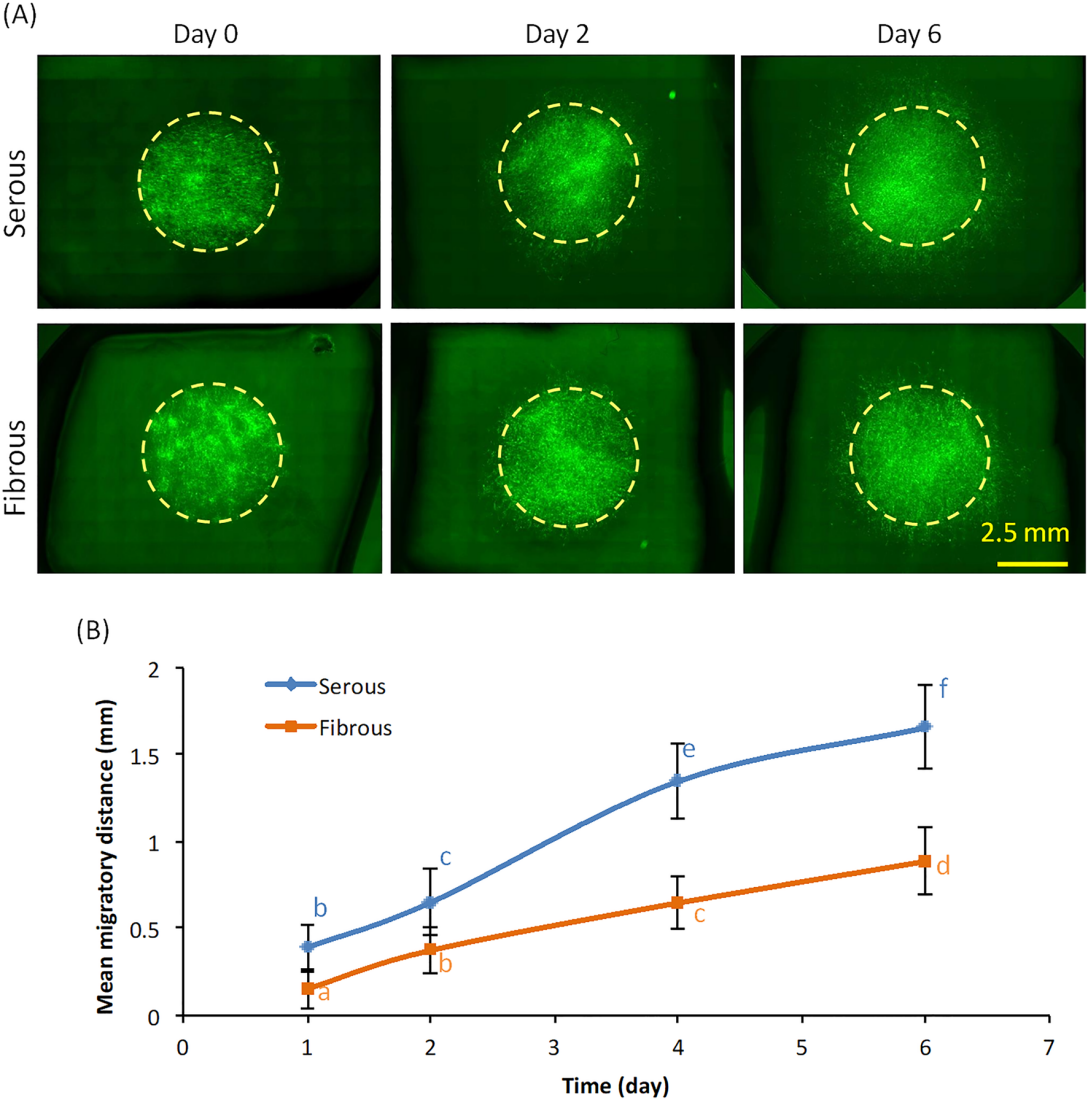
Effect of AR-BP sideness on cell migration. (**A**) Inverted microscopy images of cell migration at different time points (day 0, 2, 6), demonstrating greater cell migration following serous side seeding than fibrous side seeding. Scale bar: 2.5 mm; (**B**) Quantification of cell migration over time in culture, showing migration rate following serous seeding is greater than that of fibrous seeding. Statistical analysis was performed using two-way repeated measures ANOVA and Tukey’s multiple comparisons post hoc test. Data points that are not connected by the same lower case letter are statistically different. (n = 6 scaffolds per group).

To determine the effect of ECM niche anisotropy on cellular migration, migration of hMCS seeded onto the serous versus fibrous sides of BP-AR scaffolds was compared. Cell images were taken at specific time points (Fig. 2A) with image analysis limited to quantification of purely X–Y surface migration rate. Planar X–Y migration distance was significantly greater for hMSC seeded on the serous surface than on fibrous surface at all time points (Fig. 2B). By day 6, mean migratory distance on the serous side was 1.66 ± 0.24 mm, compared to 0.89 ± 0.19 mm on the fibrous side (p < 0.001). By day 6, mean migratory distance on serous side was 1.87 times greater than that of the fibrous side.

**Figure 3:**
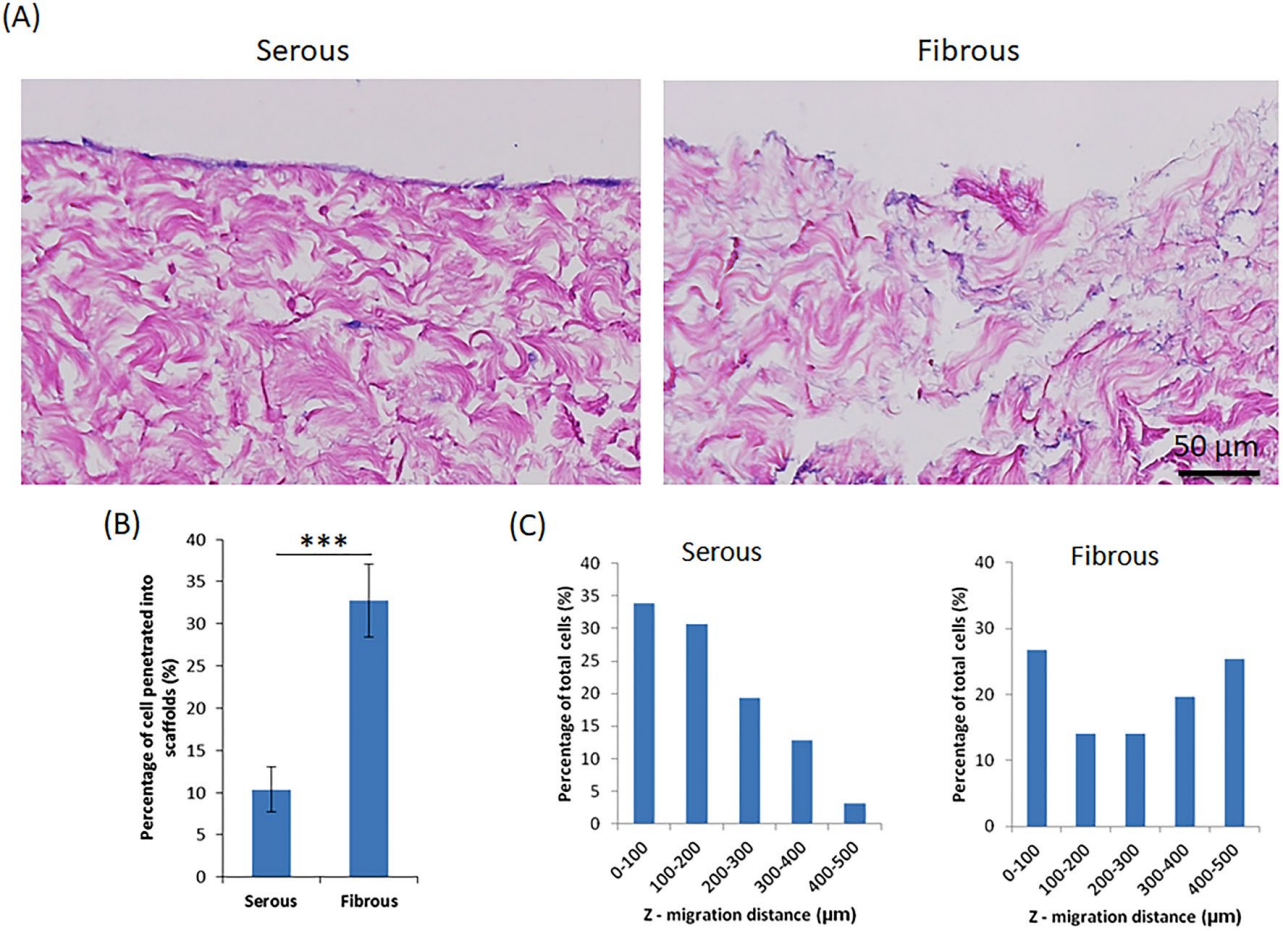
Z-direction cell migration into AR-BP scaffolds following serous or fibrous seeding. (**A**) HE images demonstrating cells remain predominantly as a monolayer on the surface following serous seeding. Conversely, cell penetration into the scaffold is higher following fibrous side seeding. (**B**) Percentage of cells penetrated into scaffolds quantified from H&E images. Significantly less cells penetrate into the scaffold following serous seeding versus fibrous seeding, p < 0.001. (**C**) Distribution of depth of penetration into the scaffold for those cells which do migrate in the Z-direction, demonstrating that overall cell penetration depth from serous side is significantly less than from fibrous side, p < 0.01. Statistical analysis was performed using one-way ANOVA, n = 6 scaffolds per group.

Despite equal seeding densities, a greater number of cells were observed on the serous surface than on fibrous surface by observing H&E staining at day 6 (Fig. 3A). Cells on serous surface formed a confluent monolayer, whereas those on the fibrous surface did not. However, a significantly higher proportion of cells penetrated in the Z-direction from the fibrous surface (32.79 ± 4.32%), compared to the serous side (10.36 ± 2.67%) (p < 0.001) (Fig. 3B,C). Furthermore, the mean Z-direction migratory distance for cells migrating from the fibrous surface (262.68 ± 166.68 µm) was significantly greater than that of cells migrating from the serous surface (180.57 ± 109.69 µm, p < 0.001) (Fig. 3C). Among all the cells penetrated inside the scaffolds, only 35% of cells migrated deeper than 200 µm from serous side; whereas 59% of cells migrated deeper than 200 µm from fibrous side.

### Effect of surface ECM niche anisotropy on cell proliferation and cell death

**Figure 4:**
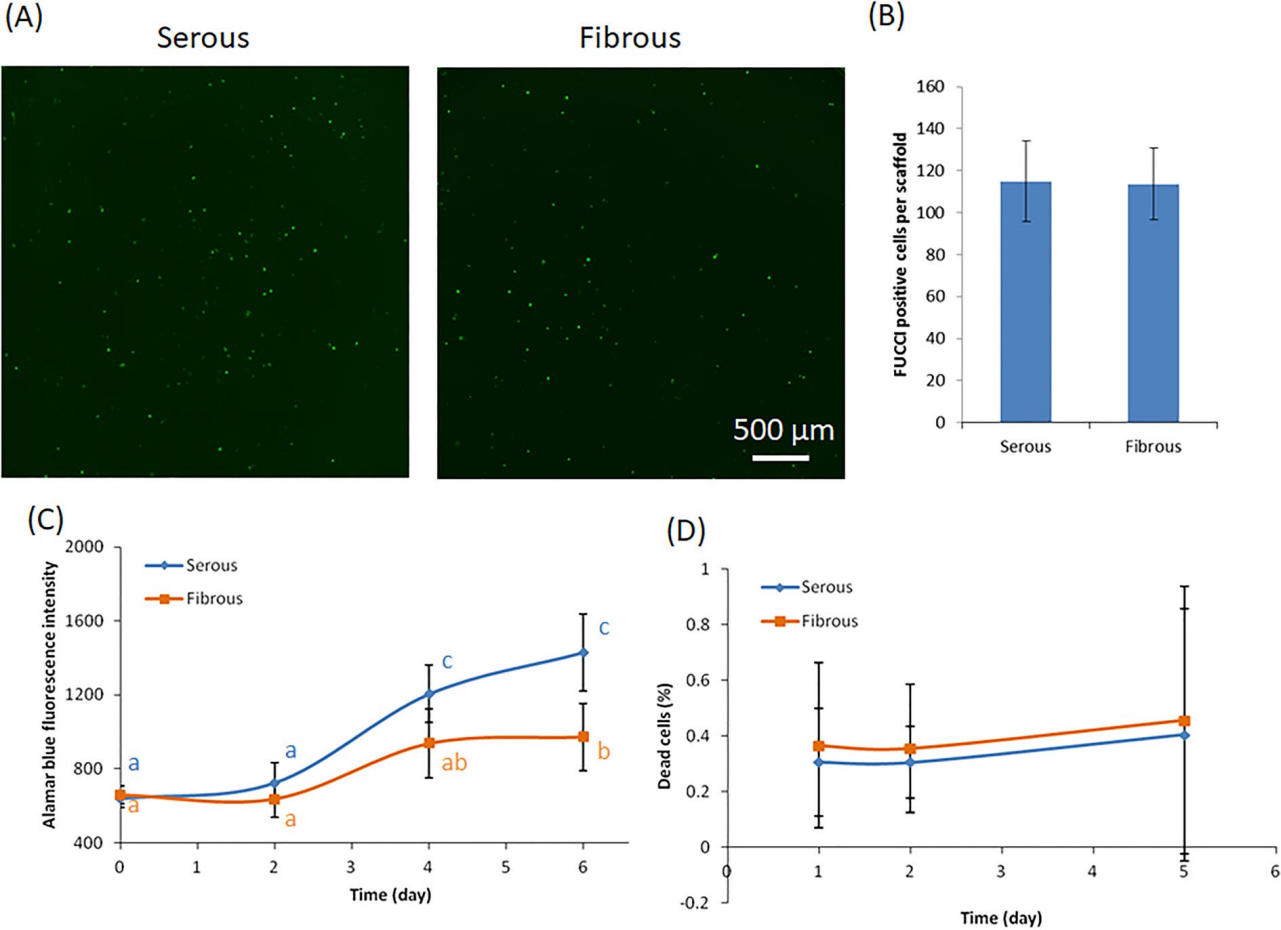
Effect of AR-BP sideness on cell proliferation. (**A**) FUCCI staining and quantification (**B**) at 6 h culture, showing no seeding side does not significantly effect number of replicating cells at early time points. Statistical analysis was performed using one-way ANOVA (n = 6 scaffolds per group). (**C**) Alarma blue intensity of cells from 0 to 6 days. At later time points (4 days and 7 days), cell number is greater following serous side seeding than fibrous side seeding. (**D**) Dead cell staining showing the dead cell percentage in both culture has no significant difference at all time points. Statistical analysis for alarma blue intensity and dead cell percentage was performed using two-way repeated measures ANOVA and Tukey’s multiple comparisons post hoc test. Data points that are not connected by the same lower case letter are significantly different. (n = 6 scaffolds per group).

Effect of ECM niche anisotropy on proliferation of hMSC was examined. Early post-seeding cell proliferation, as quantified by FUCCI staining at 6 h, did not differ between groups (Fig. 4A,B). Despite early cell proliferation identified via FUCCI, alamar blue intensity did not significantly increase in the first two days regardless of seeding surface. No significant difference in alamar blue intensity was identified between the serous and fibrous surface on days 1 and 2. Statistical difference was observed from day 3 onward, with cells seeded on the serous surface proliferating significantly more than those seeded on the fibrous surface (Fig. 4C). As a widely employed method for cell proliferation quantification, no negative effects of alamar blue on cells or scaffolds were observed. No evidence of cell death was noted in any group/time point. Percentage of dead cells was less than 0.5% throughout all culture time points, and not significant different between serous versus fibrous side seeding (p > 0.05) (Fig. 4D).

### Effect of surface ECM niche anisotropy on gene expression

**Figure 5:**
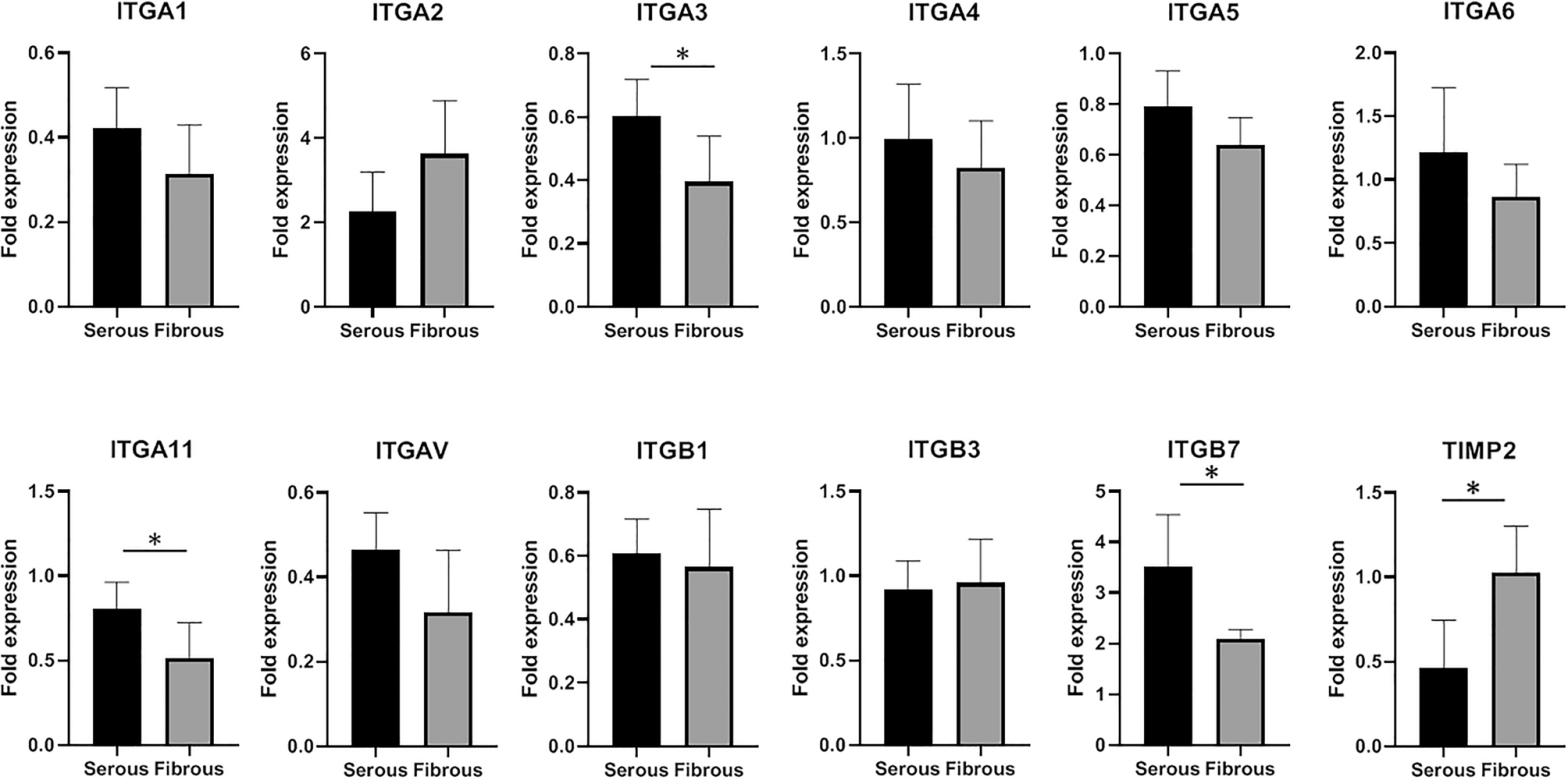
Gene analysis of cells on serous or fibrous side BP scaffold (Day 6). Expression of ITGA3, ITGA11 and ITGB7 are increased following serous side compared to fibrous side seeding. TIMP 2 expression is decreased following serous side compared to fibrous side seeding. Statistical analysis was performed using one-way ANOVA (n = 6 scaffolds per group; *p < 0.05).

To determine which integrins may be involved in the observed ECM niche mediated differential cell migration on AR-BP scaffolds, hMSCs grown on either serous or fibrous surface were collected after 6 d of culture for gene analysis. All PCR data from serous or fibrous surface seeding were normalized to cells seeded on tissue culture plastic. Major integrin genes, including α_1_, α_2_, α_3_, α_4_, α_5_, α_6_, α_11_, α_v_, β_1_, β_3_, β_7_, as well as migration-related genes such as TIMP2 and MMP2 were tested. Generally speaking, most of the integrin expression was higher in serous-hMSCs than in fibrous-hMSCs (α_1_, α_3_, α_4_, α_5_, α_6_, α_11_, α_v_, β_1_, β_3_); however, significance was only found for expression of integrins α_3_, α_11_, and β_7_ (Fig. 5). Although MMP2 (matrix metalloproteinases 2) gene, which is involved in breaking down ECM during cell migration, did not show statistically difference between serous and fibrous surface (data not shown); TIMP2, tissue inhibitor of MMP2, was significantly downregulated on serous side relative to fibrous side. These data were utilized to target candidate integrins for later antibody blocking experiments.

### Integrin blocking and gene analysis

**Figure 6:**
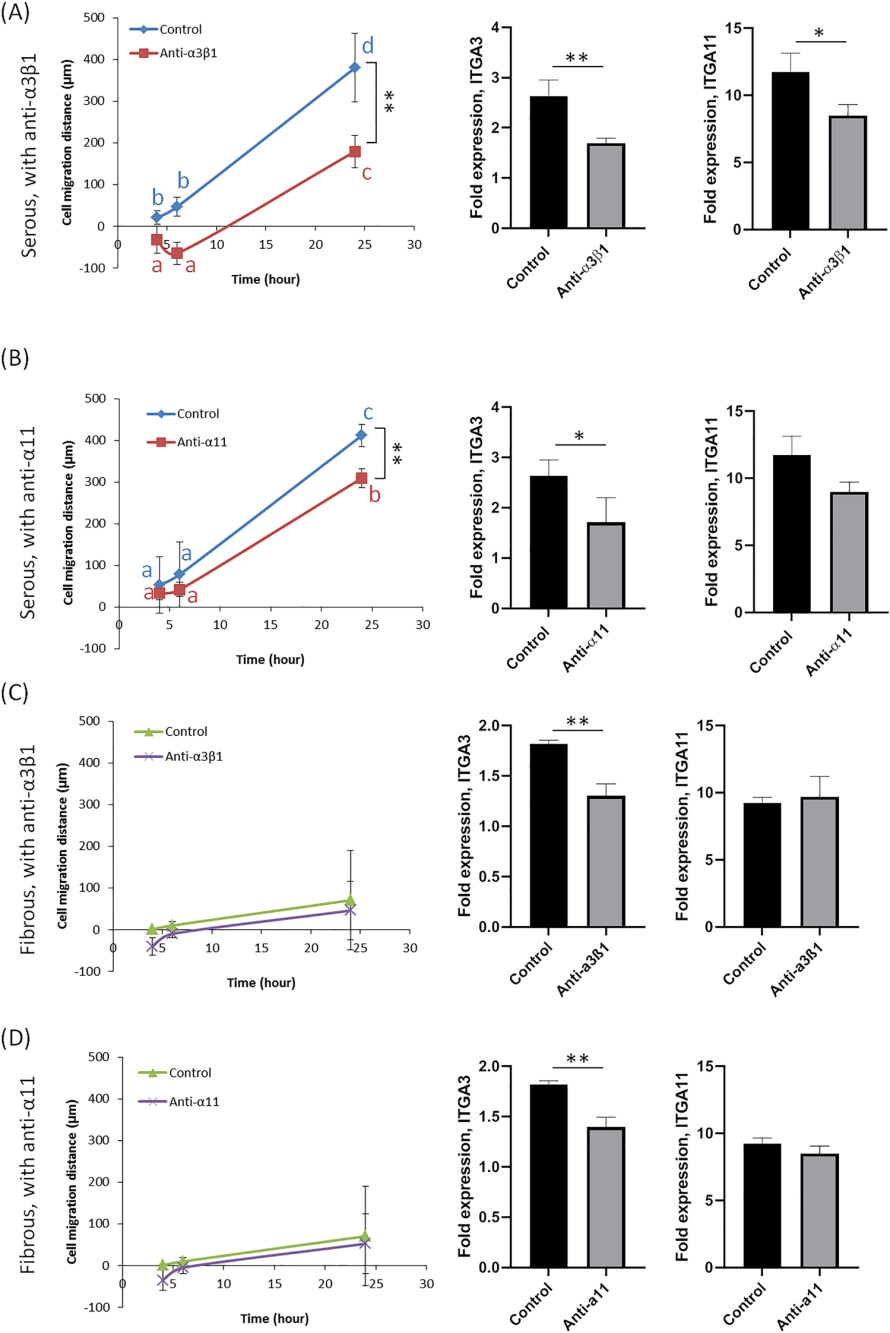
Effect of integrin blocking antibodies on cell migration and gene expression. Anti-α_3_β_1_ represents culture with integrin α_3_β_1_ antibody added; anti-α_11_ represents culture with integrin α_11_ antibody added; control represents culture without any antibody addition. (**A**) Addition of anti-integrin α_3_β_1_ (20 µg/mL) on serous side cell culture, significantly reduces cell migration. Additionally, expression of ITGA3 and ITGA11 are reduced following anti-integrin α_3_β_1_ antibody blockade. (**B**) Addition of anti-integrin α_11_ (20 µg/mL) on serous-side cell culture, significantly reduces cell migration only at later time points (i.e., 24 h). Expression of ITGA3, is reduced following anti-integrin α_11_ antibody blockade. (**C**) Addition of anti-integrin α_3_β_1_ (20 µg/mL) on fibrous side cell culture results in no significant difference in cell migration. Expression of ITGA3 is reduced following anti-integrin α_3_β_1_ antibody blockade. (**D**) Addition of anti-integrin α_11_ (20 µg/mL) on fibrous-side cell culture results in no significant difference in cell migration. However, expression of ITGA3 is reduced following anti-integrin α_11_ antibody blockade. Statistical analysis was performed using one-way ANOVA with repeated measures for time series data (n = 6 scaffolds per group; *p < 0.05; **p < 0.01). Data points that are not connected by the same lower case letter are significantly different.

In order to show that integrin α_3_β_1_ (laminin binding) and integrin α_11_β_1_ (Col IV binding) are one of the potential mechanisms involved in mediating cell migration on basement membrane (Serous) versus non-basement membrane (Fibrous) ECM environments, integrin blocking experiment were performed. Addition of anti-integrin α_3_β_1_ and anti-integrin α_11_ into cell culture medium significantly decreased cell migration on the serous side (Fig. 6). Blockade with anti-integrin α_3_β_1_ antibody prevented early migration (i.e., 4 and 6 h) on the serous surface, although migration resumed at later time points (Fig. 6A). Conversely blockade with anti-integrin α_11_ antibody inhibited serous side migration after the 6 h time point (Fig. 6B). By 24 h, mean cell migratory distance on the serous surface decreased by 52.8 ± 10.3% (anti-α_3_β_1_) and 24.8 ± 0.8% (anti-α_11_) respectively, in the presence of integrin blockade. Conversely, addition of anti-integrin antibodies did not change cell migration significantly on the fibrous surface (Fig. 6C,D).

With addition of anti-integrin α_3_β_1_ to cell culture on serous surface, integrin α_3_ and α_11_ gene expression significantly decreased. Blockade with anti-integrin α_11_β_1_ to cell culture on serous surface only significantly reduced integrin α_3_ expression and had no effect on α_11_ gene expression. For cells cultured on fibrous surface, addition of anti-integrin α_3_β_1_ or anti-integrin α_11_β_1_ significantly decreased integrin α_3_ expression only, and had no effect on α_11_ expression.

## Discussion

The present study demonstrates the importance of different ECM niche (i.e., basement membrane versus non-basement membrane) on hMSCs migration. Specifically, we compared the effect of basement membrane presence (serous side seeding) versus absence (fibrous side seeding) on hMSC behavior and found that: (1) AR-BP preserved basement membrane proteins laminin and Col IV on serous surface; (2) hMSCs migrated rate is increased following seeding on the basement membrane containing surface (i.e., serous side) of AR-BP scaffold compared to the non-basement membrane surface (i.e., fibrous side); (3) basement membrane presence prevents Z-direction migration of cells seeded on BP-AR scaffolds; (4) higher expression of integrins α_3_ and α_11_ from cells seeded on the serous surface are two of the major mechanism for enhanced cell migration. (5) Blockade of integrin α_3_β_1_ activity inhibits early post-seeding migration, while blockade of integrin α_11_β_1_ inhibits late cell migration on basement membrane containing surfaces. These findings demonstrate that laminin binding via integrin α_3_β_1_ and Col IV binding via integrin α_11_β_1_ are major mechanisms for early and late hMSC migration respectively, following seeding on basement membrane containing surfaces.

Basement membranes are a critical component in ECM niche of vascular tissue that supports and facilitates cell growth and function. However, most commonly used decellularization reagents such as sodium dodecyl sulfate (SDS), sodium deoxycholate, 3-[(3-cholamidopropyl) dimethylammonio]-1-propanesulfonate (CHAPS) have been shown to disrupt basement membrane structure and composition in ECM scaffolds^7^. Disruption and/or denaturing of native basement membrane structure can negatively impact the cell interaction with ECM scaffolds. Indeed, previous studies have demonstrated reduced cell viability when seeded onto SDS-decellularized bovine pericardium; even after 7 days washout, the toxicity of SDS scaffold remained at 96.5%^15^. Furthermore, ECM mediated differential cell behavior is abolished in SDS-decellularized BP^15^. The current study demonstrates that AR-BP maintains basement membrane integrity (i.e., laminin and collagen IV organization and content).

Various studies have shown that individual ECM molecules, especially basement membrane components Col IV and laminin, can promote cell adhesion and migration in vitro. For instance, coating fibronectin, Col IV or laminin on polycarbonate filters promotes lens epithelial cell migration in a concentration dependent manner^16^. Collagen IV coating on cell culture plates has also been shown to increase aortic endothelial cell adhesion and migration^17^. Laminin and fibronectin can stimulate directional migration of B16 murine metastatic melanoma cells in vitro as assessed in modified Boyden chambers^18^. However, these studies only investigated the effects of single component on cell migration. In this work, we used AR-BP scaffold which maintained major basement membrane components on serous surface, to allow investigation of the impact of the complex ECM niche on cell migration. We found that hMSCs cultured on serous surface migrated significantly faster than cells on fibrous surface. Our results are consistent with previous studies that presence of individual basement membrane components promote cell migration.

Cell migration is a complex process, and multiple independent mechanism may be involved in cell response to ECM proteins. Integrins play a critical role in mediating cell adhesion and migration^19^. Comparing integrin gene expression of hMSCs cultured on serous and fibrous surface, we found that serous-seeded hMSCs expressed significantly higher level of integrin α_3_ and integrin α_11_ than fibrous-seeded hMSCs. Integrin dimer α_3_β_1_ mainly binds to laminin, so the presence of laminin on the serous surface may explain the enhanced expression of this laminin binding integrin. Comparing gene expression of integrin α_3_ and α_11_ from cells grown on serous surface on day 1 (Fig. 6) and day 6 (Fig. 5), the relative expression (normalized to tissue culture plastic) is much higher on day 1. This observation might indicate that cells were making a lot more integrin at early adhesion and migration stage in order to move around aggressively, but making less integrin at later time points because they had already produced sufficient integrin to achieve their matrix mediated migration rate. It was reported that loss of integrin α_3_β_1_ altered actin cytoskeleton dynamics at the leading edge of migrating neurons leading to cell migration defect^20^. Conversely, integrin dimer α_11_β_1_ is a receptor for collagen, including Col I and Col IV, and supports cell migration through collagen rich ECM. Previous studies demonstrated that integrin α_11_β_1_ mediated cell adhesion to collagens I and IV and formed focal contacts on collagens^21^. Blocking integrin α_3_β_1_ and α_11_β_1_ by antibodies significantly decreased cell migration on serous surface within 24 h, which confirmed that these two integrins are involved in hMSCs migration on serous surface. Importantly, the effect of integrin α_3_β_1_ blockade was most prominent at early time points (4 and 6 h) whereas integrin α_11_β_1_ blockade prevented migration only at later time points (24 h). However, blocking integrin α_3_β_1_ and α_11_β_1_ did not have significant impact on cell migration on fibrous surface, which were probably due to the lack of laminin and collagen IV on fibrous surface. Addition of anti-integrin α_3_β_1_ antibody on serous-hMSCs significantly decreased integrin α_3_ and α_11_ gene expression; addition of anti-integrin α_11_β_1_ antibody on serous-hMSCs only significantly decreased α_3_ expression. These results suggested that integrin α_3_ is more critical than integrin α_11_ in directing cell migration on basement membrane. Previous studies have shown that laminin is the most important basement membrane constituent directing epithelial cell migration^16^. Comparison between laminin and Col IV demonstrated that laminin is more effective in promoting cell migration; collagen IV is more effective in promoting cell adhesion^16^. Another study found that among various matrix proteins laminin-5 was most potent in promoting adhesion and migration of different kinds of glioma cells; and integrin α_3_β_1_ specifically mediated the interaction with laminin-5^22^. On the fibrous surface, addition of anti-integrin α_3_β_1_ antibody did not block cell migration, although integrin α_3_ gene expression significantly decreased. Similarly, addition of anti-integrin α_11_β_1_ antibody did not block fibrous side cell migration despite significantly decreasing integrin α_3_ gene expression. In both case, integrin α_11_ gene expression did not change significantly. These results suggested that neither integrin α_3_ or integrin α_11_ are critical regulators of cell migration on the non-basement membrane surface (i.e., Col I) of ECM scaffolds.

## Conclusions

This study demonstrates differential hMSCs migration behavior on serous and fibrous surface of AR-BP ECM scaffolds. AR-BP scaffolds maintained major basement membrane proteins laminin and Col IV on serous surface, which significantly promoted planar cell migration; whereas, the fibrous surface does not contain laminin and Col IV and consequently modulates slower hMSCs planar migration. Conversely, Z-direction migration is higher in the non-basement membrane environment than in the basement membrane containing ECM niche. Furthermore, early hMSCs migration on serous surface is predominantly mediated through integrin α_3_β_1_ interaction with laminin, while later hMSC migration is mediated via integrin α_11_β_1_ interaction with Col I. These findings are valuable for selection of the optimal surface of BP scaffold for clinical applications. Serous side of AR-BP stimulates faster cell migration and resultant monolayer formation than fibrous side; therefore, it is reasonable to orient the serous surface toward the vascular lumen when considering utilization of AR-BP as a vascular patch.

## Methods

### BP scaffold preparation

Bovine pericardial sacs from adult cattle (Spear Products, Coopersburg, PA) were stripped of epicardial fat and loose connective tissue. Pericardium was cut into square pieces (1 × 1 cm), and subjected to AR as previously described^11^. Briefly, BP pieces were subjected to hydrophile solubilization (10 mM Tris–HCl pH 8, 0.5 mM Pefabloc, 100 mM DTT, 100 mM KCl, 2 mM MgCl_2_·6H_2_O, 1% (v/v) antibiotic antimycotic solution, Sigma) for 48 h, followed by lipophile solubilization (3% (w/v) ASB-16 in hydrophile solubilization buffer) for 48 h at room temperature. Then all samples were treated by nucleic acid digestion (10 mM Tris–HCl pH 7.6, 150 mM NaCl, 5 mM MgCl_2_·6H_2_O, 2.5 Kunitz units/mL DNAse I, 7.5 Kunitz units/mL RNAse A, 1% (v/v) antibiotic antimycotic solution) for 24 h, followed by washout in 0.5 mM Pefabloc, 10% (v/v) Tris-Buffered saline, 1% (v/v) antibiotic antimycotic solution for 96 h. All the solutions were changed twice a day.

### Cell seeding and culture

hMSC were isolated from fresh, unprocessed human bone marrow from three healthy donors (Lonza, Allendale, NJ) and transduced with enhanced green fluorescent protein (eGFP) as previously described^15^. All hMSCs employed in these studies have been previously qualified for stemness by trilineage differentiation and surface marker expression studies^15,23^. For cell seeding experiments, BP-AR scaffolds were placed in the bottom of 24-well plates with either the serous or fibrous surface face up. 5 mm glass cylinders were placed on the center of each scaffold and used to confine seeding of eGFP-hMSCs at a density of 500 cells/mm^2^ in 100 µL hMSC culture media (Dulbecco’s modified Eagle’s medium high glucose (DMEM), 1% (v/v) penicillin–streptomycin (P/S), 1% (v/v) l-glutamine 200 mM (Hyclone Laboratories, South Logan, UT, USA) and 10% (v/v) fetal bovine serum (FBS, Atlanta Biologicals, Lawrenceville, GA, USA). Glass cylinders were removed after overnight incubation (Day 0). Seeded scaffolds were cultured at 37 °C and 5% CO_2_. Culture media was changed daily.

### Quantification of X–Y cell migration distance

Cells were imaged on day 0, 1, 2, 4, and 6 using Nikon inverted fluorescence microscopy at 4× magnification, with the entire scaffold surface imaged using an automated stage and stitching software (NIS Elements, Nikon). The cell migration distance was defined as the mean migratory distance which was obtained by calculating total area covered by cells, converting this area to that of a circle of equivalent size and employing the radius of this circle as a non-directional estimate of mean migratory distance at each time point. The increase in mean migratory distance was compared to initial seeding location (i.e., radius at each time point minus radius at time 0). Six scaffolds were analyzed for each group.

### Proliferation

Seeded scaffolds were cultured for 6 days, with alamar blue assay performed on days 0, 2, 4, and 6. Alamar blue reagent was mixed with cell culture media at the ratio of 1:10. Cells on scaffolds were washed with fresh media and incubated in 250 µL alamar blue reagent mixture for 1.5 h at 37 °C. 100 µL of supernatant from each well was transferred in 96-well plate for measurement of fluorescence intensity using a Cytation3 imaging reader (560Ex/590Em). Premo FUCCI cell cycle experiment was performed according to manufacturer’s recommendations. Briefly, cells plated on tissue culture flask were transfected with Premo reagent. After overnight incubation, cells were harvested and seeded on either serous or fibrous AR-BP scaffolds. After 6 h, the total fluorescent cells on each scaffold were counted. The scaffolds were imaged using Nikon inverted fluorescence microscopy at 4× magnification, with the entire scaffold surface imaged using an automated stage and stitching software. Six scaffolds were analyzed for each group.

### Dead cell assay

Seeded scaffolds were cultured for 5 days, with dead cell assay performed on days 1, 2 and 5. Scaffolds were washed with 1 mL of phosphate-buffer saline (PBS, Sigma) to remove any cell debris and incubated for 5 min in 1 mL of ethidium homodimer (6 µM, Sigma) in PBS. After the incubation, the ethidium homodimer solution was removed and replaced with 1 mL of cell media. The scaffolds were imaged using Nikon inverted fluorescence microscopy at 4× magnification, with the entire scaffold surface imaged using an automated stage and stitching software. The red fluorescent cells (dead cells) were counted using MATLAB and expressed as a ratio of green fluorescent live cells number. Six scaffolds were analyzed for each group.

### Quantification of cell Z-direction migratory distance

The H&E histology section of day 6 cell-seeded BP scaffolds were imaged using Nikon microscopy. The Z-direction migratory distance was defined as the shortest distance from the cell to the seeding surface. Percentage of cells penetrated into scaffold was defined as ratio between cells with migratory distance larger than 0 and all cells counted in each image. The penetration depth distribution only accounted cells with migratory distance larger than 0. The percentage of total cells (Fig. 3C) was defined as ratio between cells with specific migratory distance and all cells penetrated inside scaffolds. Six scaffolds were analyzed for each group and three random power fields were analyzed for each scaffold.

### Immunohistochemistry and immunofluorescence staining

Histology sections of BP scaffolds with or without hMSCs seeding were processed using H&E staining, and immunofluorescence staining. Briefly, scaffolds were fixed in 10% buffered formalin, embedded in paraffin, and 4 µm sections created for staining. Immunofluorescence staining for basement membrane proteins laminin and collagen IV was performed using rabbit anti-laminin (1:50, Invitrogen,) and rabbit anti-collagen IV (1:200, Abcam, Cambridge, MA, USA) primary antibody. Fluorescent anti-rabbit secondary antibody tagged with Alexa Fluor 647 or Alexa Fluor 488 (1:200, Abcam) was used for visualization. Nikon Eclipse Ni-E microscopy was used to image H&E slides under 20× magnification and fluorescence staining slides under 40× magnification.

### RT-PCR

After 6 days of culture, total RNA from seeded scaffolds were extracted using the RNeasy Mini plus kit (Qiagen, Valencia, CA). Primers specific for target genes (Supplementary Table 1) were purchased from IDT and GAPDH was used as housekeeping gene. Cells cultured on tissue culture plastic were used as control. RT-PCR reactions were performed on QuantStudio 7 Flex real-time RT-PCR system (Applied Biosystems, Foster City, CA, USA), using Tagman PCR Master Mix. The amplification reactions were carried out for up to 40 cycles. Fold variation in gene expression was quantified using the comparative Ct method: 2^−(ΔCtTreatment−ΔCtControl)^.

### Integrin blocking experiment

Integrin blocking experiments were performed by adding anti-integrin α_3_β_1_ (20 µg/mL) or anti-integrin α_11_ (20 µg/mL) antibody (Abcam, MA) to cell culture medium. Cells were cultured up to 24 h and cell migration was imaged at 0, 4, 6 and 24 h. Then total RNA was extracted for gene analysis.

### Statistical analysis

All data are expressed as mean ± standard deviation. One-way ANOVA were performed on gene analysis, FUCCI, and Z-migration experiment. Two-way repeated measures ANOVA and Tukey’s multiple comparisons post hoc test were performed on average mean migratory distance, alamar blue proliferation, and dead cell experiments. For all analyses p < 0.05 was considered to be significant.

## Supplementary Information

Supplementary Table 1.
