# Supplementary material for: Basement membrane proteins modulate cell migration on bovine pericardium extracellular matrix scaffold
Source: Sci Rep. 2021 Feb 25;11:4607. doi: 10.1038/s41598-021-84161-5 (PMC7907089; doi:10.1038/s41598-021-84161-5)
Supplement: Supplementary file 1 — Supplementary Table 1. [file 41598_2021_84161_MOESM1_ESM.docx]

Basement Membrane Proteins Modulate Cell Migration on Bovine Pericardium Extracellular Matrix Scaffold

Qi Xing, Mojtaba Parvizi, Manuela Lopera-Higuita, Leigh G. Griffiths^*^

Depart of Cardiovascular Diseases, Mayo Clinic, 200 First Street, Rochester, MN, 55905

Corresponding author:

Leigh G. Griffiths, Email: [Griffiths.Leigh@mayo.edu](mailto:Griffiths.Leigh@mayo.edu), Mailing address: Stabile 4-58, Mayo Clinic, 200 First Street, Rochester, MN, 55905

**Supplementary Table 1**. Primers and probe sequences for the genes studied.

| Gene | **Primer 1** | **Primer 2** | **Probe sequence** |
| --- | --- | --- | --- |
| *GAPDH* | TGTAGTTGAGGTCAATGAAGG G | ACATCGCTCAGACACCATG | AAGGTCGGAGTCAACGGATTTGGTC |
| *ITGA1* | ACTCTCCATCTGTCACAATAACC | GCCAGACTATGACAGCTCTTG | TGAATGCCTCCTTTCTTGCTGTGTCT |
| *ITGA2* | AACCTCCAGTTCCCATGT TC | AATGTCCTGTTGACCTATCCAC | TGGTGAGGATCAAGCCGAGGC |
| *ITGA3* | CCCTTAGAGCTACTGTGATAGATG | CAGCATTGGTGACATCAACC | CCGTTTGAAGGCTTGGGCAAAGT |
| *ITGA4* | AACCCATGAACAGTCAGCTTA | CTCAGTATCACAGTGCATGCTA | TGCTACAGTCACTCTGCTGTGCTTT |
| *ITGA5* | TTGTACACAGCCTCACACTG | ACCAACAAGAGAGCCAAAGTC | TGCTCCCGCTGCAAGAAAGTCT |
| *ITGA6* | CATATCGGTGAGCACATGTCA | TCGAGTTTGATAACGATGCTGA | CCC CATCCACTGATCTTCCTTGCTT |
| *ITGA11* | GTCCCGCTCATTACTGTCAC | AAGTCTGCAACGTCAGCTATC | AGCAAATCCATCTTCCTACACCACCTG |
| *ITGAV* | AAAGTCATCTATGCCATCACCA | ACTGCACAAGCTATTTTTGATGAC | CTCCGACAGCCACAGAATAACCCAA |
| *ITGB1* | GGTCAATGGGATAGTCTTCAGC | GTAGCAAAGGAACAGCAGAGA | AGCCAGAGGATATTACTCAGATCCAACCA |
| *ITGB2* | TCACTCCATTGCTGCAGAAG | AGCTGTCTGAGGACTCCAG | CCCGACACCCTGAAAGTCACCT |
| *ITGB3* | CCCTTAGAGCTACTGTGATAGATG | CAGCATTGGTGACATCAACC | CCGTTTGAAGGCTTGGGCAAAGT |
| *ITGB7* | CCTTACCCTCCCTCTTCTCA | GTGGTACAGCTCATCATGGA | TGACCCTTGAACACTCTTCACTCCCT |
| *MMP2* | GTGCAGCTGTCATAGGATGT | TCCACCACCTACAACTTTGAG | TTCTGTCCCCATGAAGCCCTGTTC |
| *TIMP2* | TGTGGTTCAGGCTCTTCTTC | GACGTTGGAGGAAAGAAGGA | TCTCATTGCAGGAAAGGCCGAGG |
